# Multifocal Oral Epithelial Hyperplasia Associated With Papillomavirus Infection in a Road Killed Brown Howler Monkey (*Alouatta guariba clamitans*)

**DOI:** 10.1111/jmp.70102

**Published:** 2026-08-04

**Authors:** Sandy Lorena Pulecio‐Santos, Vivian Renata Kida, Alex Junior Souza de Souza, José Luiz Catão‐Dias, Pedro Enrique Navas Suarez, Lilian Rose Marques de Sá

**Affiliations:** ^1^ Department of Pathology, School of Veterinary Medicine and Animal Science University of São Paulo (USP) São Paulo Brazil

**Keywords:** emergent virus, neotropical primates, pathology, primates virology, wildlife

## Abstract

A free‐ranging road‐killed brown howler monkey ( *Alouatta guariba clamitans* ) presented multiple mucosal‐colored papules and plaques on oral mucosa. Pathological evaluation confirmed the diagnosis of epithelial hyperplasia with koilocytosis. Papillomavirus antigens were demonstrated by immunohistochemistry, and PCR was positive for partial amplification of *Alouatta guariba* papillomavirus 1 DNA.

## 1 Introduction

Papillomavirus (PV) is a host‐specific virus that has been described in fish, reptiles, birds, and mammals, such as nonhuman primates (NHP), hominids, and humans [1, 2]. PV infection is a recognized oncogenic driver, associated with the development of benign and malignant tumors, as well as oral focal epithelial hyperplasia [3, 4, 5, 6]. PV has been isolated from the oral mucosa of hominids, such as bonobos ( *Pan paniscus* ) and chimpanzees ( *Pan troglodytes* ); from the cervicovaginal and genital mucosa of Old World monkeys, including long‐tailed macaques ( *Macaca fascicularis* ) and rhesus macaques ( *Macaca mulatta* ); and from the forehead skin of Strepsirrhines, such as ruffed lemurs ( *Varecia variegata variegata* ) [7, 8, 9, 10, 11, 12]. In New World Primates (NWP), reports include isolates from skin and anal swabs in spider monkeys ( *Ateles geoffroyi* ) and black‐tufted marmosets ( *Callithrix penicillata* ), oral and genital swabs from black‐and‐gold howler monkeys ( *Alouatta caraya* ), azara's capuchins ( *Sapajus cay* ), and black capuchins ( *Sapajus nigritus* ), and from the oral mucosa of brown howler monkeys ( *Alouatta guariba clamitans* ) [13, 14, 15, 16]. Nevertheless, several reports indicate that PV isolation in these species is not always associated with the presence of clinical lesions [5, 11, 13, 14, 17, 18].

The first identification of PV in NWP was reported in a free‐ranging brown howler monkey ( *Alouatta guariba clamitans* ) presenting with oral focal epithelial hyperplasia (FEH) in Brazil [19]. The first complete genome sequence of a PV in NWP was subsequently characterized and designated as *Alouatta guariba* papillomavirus 1 (AgPV1) [15]. Despite these initial findings, comprehensive case reports and diagnostic characterizations remain scarce. Howler monkeys are one of the largest Neotropical primate species in the Brazilian Southeastern region and are currently classified as “Vulnerable” in terms of their conservation status [20]. This report aims to characterize and support the diagnosis of PV associated with oral lesions in a howler monkey, contributing to the understanding of this process and aiding in the dissemination of its diagnosis in NWPs.

## 2 Case Report

A free‐ranging, male, aged adult brown howler monkey was referred to necropsy. The animal was found dead due to vehicle collision on highway BR116 in the municipality of Embu, São Paulo State (23°40′16.705″S; 46°51′21.989″W).

**FIGURE 1:**
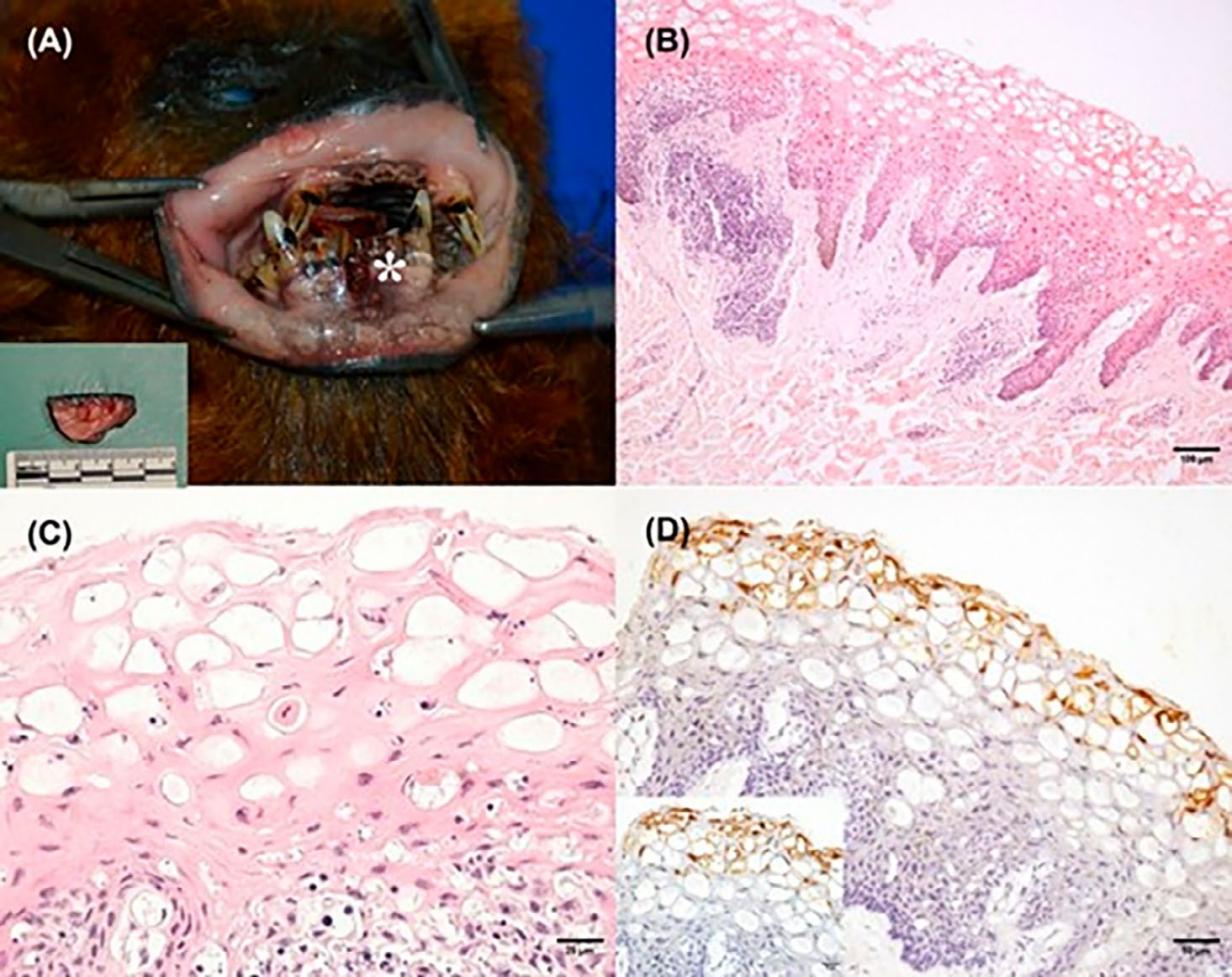
Oral multifocal epithelial hyperplasia, brown howler monkey ( *Alouatta guariba clamitans* ). (A) Mucosal surface of the upper and lower lips presented multifocal and coalescent plaque lesions, diffuse irregular gingival surface, some with melanin pigment, and other mucosa colored. Note the mandibular fracture (*). Inset: The upper lips presented flat wart‐like structures, well delimited with mucosal‐colored; (B) Oral mucosa. Moderate hyperplasia of the basal layer, tortuous epithelial cones, and inflammatory infiltrate in the submucosa. H&E. Bar 100 μm; (C) Oral mucosa. Koilocytosis and dyskeratosis in the spinous stratum. H&E. Bar 25 μm; (D) Multiple keratinocytes positive to human papillomavirus antibody in the nucleus by immunohistochemistry, DAB as chromogen. Harris hematoxylin as counterstain. Bar 50 μm. Inset: Bar 25 μm.

At gross, autolysis was moderate, it was in good corporal condition (6.0 kg), there were multiple foci of cutaneous abrasion associated with multiple subcutaneous hematomas on the right side of the thoracoabdominal area, and marked hemothorax, which was considered the cause of death. The lower lip mucosa presented multifocal and coalescent, discrete elevated plaque and flat wart‐like lesions well delimited with a minimum diameter of 0.2 cm up to 0.7 cm, and mucosa‐colored (Figure 1A) associated with a diffuse, pigmented gingival surface. Samples of oral mucosa were collected, frozen at −20°C, fixed in 10% formalin, histologically processed, and stained with hematoxylin and eosin for microscopical examination.

The histopathological evaluation revealed a mild freezing artifact related to deformed keratinocytes in the spinous stratum. The oral lesions were characterized by marked irregular hyperplasia with bridge fusion associated with moderate irregular hyperplasia of the spinous stratum. Binucleation, dyskeratosis, and koilocytosis in keratinocytes of the spinous stratum were multifocal to focally extensive in the epithelial surface. There was a moderate perivascular inflammatory infiltrate of lymphocytes, plasma cells, and a few macrophages in the submucosa (Figure 1B,C).

The PV antigens were detected by immunohistochemistry (Figure 1D), using a primary anti‐human papillomavirus monoclonal antibody (cod. M3528, clone K1H8, dilution 1/1000, Dako, USA), without antigenic recovery. The samples were processed using system K0690 (Dako, USA) for 30 min at 37°C in a humid chamber. Diaminobenzidine solution (DAB, cod. D5637, Sigma) and Harris hematoxylin (Merck, USA) were used as chromogen and counterstain, respectively. Histological section of oral mucosa from a previous case of FEH in brown howler monkey, confirmed for AgPV1 infection, was used as positive controls. Oral section without the addition of primary antibody was used as negative control.

**FIGURE 2:**
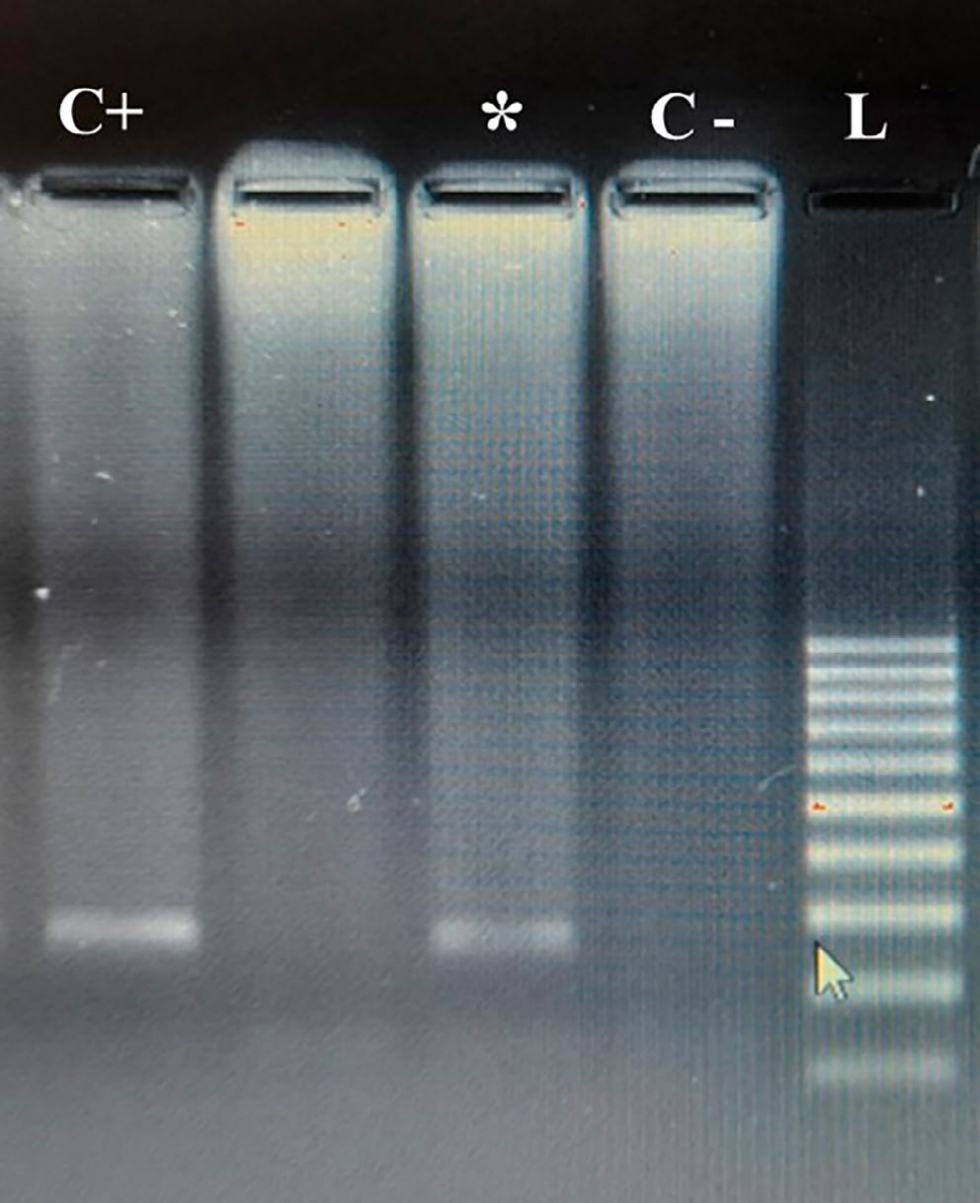
Agarose gel electrophoresis (1.5%) for detection of *Alouatta guariba clamitans* Papillomavirus type 1 DNA. Lane C+: Positive controls showing a specific band at approximately 300 bp. Lane *: Positive sample demonstrating a band corresponding to the expected size of 300 bp. Lane L: 100 bp DNA ladder. Lane N: Negative control.

An oral fragment was subjected to DNA extraction and purification with the DNeasy Blood & Tissue kit (QIAGEN). The DNA was applied in a conventional PCR assay for partial amplification of the L1 gene (~300 bp) of AgPV1 using the Platinum II Taq Hot‐Start DNA Polymerase kit (Thermo Fisher Scientific) and primers AgPV_F6873 (5′ ATACTACCCGCAGCACCAAT 3′) and AgPV_R7174 (5′ CTCCTTAGGGGGAACCTTGG 3′) under the following thermocycling program: initial denaturation at 94°C for 5 min; 35 cycles of denaturation at 94°C for 30 s, annealing at 58°C for 30 s, extension at 72°C for 45 s; and final extension at 72°C for 10 min. The design of primers for specific amplification of AgPV1 was based on complete viral genome sequences, and the PCR protocol was previously validated with samples from brown howler monkeys positive for viral infection [15]. The amplicons were detected in 1.5% agarose gel electrophoresis, and the sample obtained in this case presented positive amplification for AgPV1 infection (Figure 2).

## 3 Discussion

This study characterizes oral lesions associated with AgPV1. Recognizing gross and microscopic presentations is essential for diagnosing FEH in NWPs, especially as PV infections in other species often lack clinical lesions and are associated with latency or oncogenesis [21, 22, 23, 24]. Currently, AgPV1‐associated FEH has only been diagnosed in brown howler monkeys, highlighting its host specificity.

The FEH features described were consistent with active PV infection and inflammatory response, as previously reported [19]. In humans, this condition is primarily associated with human papillomavirus (HPV)‐13 and HPV‐32 and has been described predominantly in indigenous communities [25, 26, 27, 28, 29], including recent reports in Brazilian indigenous children [30].

PV‐associated lesions in humans have been linked to immunosuppression and a higher incidence among genetically related individuals [3, 22, 27, 31, 32, 33]. Notably, the howler monkey was a free‐ranging, aged adult animal with no gross evidence of immunosuppression or sickness. This finding, alongside previous similar cases [34], provides critical evidence regarding the circulation of AgPV1 within the state of São Paulo and its metropolitan region, demonstrating a presentation that is consistent with findings previously described in other howler monkeys [19, 34].

Monitoring and reporting emerging infectious agents serve as a vital tool for clinical practice and wildlife conservation efforts, including surveillance in road‐killed NWPs. Despite their importance, reports of PV infection in this species or other NWP species remain too scarce to determine host distribution, potential comorbidities, the presence of pre‐existing conditions, tissue response, or a specific predisposition to secondary diseases.

## Funding

This study was partially funded by the Coordenação de Aperfeiçoamento de Pessoal de Nível Superior—CAPES, with the study grant awarded (Grant number: 88887.682555/2022‐00).

## Ethics Statement

The present study was conducted in accordance with the legal requirements for the use of Brazilian genetic heritage. All procedures and data access were registered in the National System for the Management of Genetic Heritage and Associated Traditional Knowledge (SisGen) under registration number (A16D32C). Sample collection was previously authorized by the Chico Mendes Institute for Biodiversity Conservation (ICMBio) through the Biodiversity Authorization and Information System (SISBIO), license number 85398‐1. The Ethics Committee on the Use of Animals (CEUA) of the FMVZ—USP approved the study under the number 4103250222.

## Conflicts of Interest

The authors declare no conflicts of interest.

## Data Availability

The data that support the findings of this study are available on request from the corresponding author. The data are not publicly available due to privacy or ethical restrictions.
